# Fast-Dissolving Core-Shell Composite Microparticles of Quercetin Fabricated Using a Coaxial Electrospray Process

**DOI:** 10.1371/journal.pone.0092106

**Published:** 2014-03-18

**Authors:** Chen Li, Deng-Guang Yu, Gareth R. Williams, Zhuan-Hua Wang

**Affiliations:** 1 Institute of Biotechnology, Shanxi University, Taiyuan, China; 2 School of Materials & Engineering, University of Shanghai for Science and Technology, Shanghai, China; 3 UCL School of Pharmacy, London, United Kingdom; University of California, Merced, United States of America

## Abstract

This study reports on novel fast-dissolving core-shell composite microparticles of quercetin fabricated using coaxial electrospraying. A PVC-coated concentric spinneret was developed to conduct the electrospray process. A series of analyses were undertaken to characterize the resultant particles in terms of their morphology, the physical form of their components, and their functional performance. Scanning and transmission electron microscopies revealed that the microparticles had spherical morphologies with clear core-shell structure visible. Differential scanning calorimetry and X-ray diffraction verified that the quercetin active ingredient in the core and sucralose and sodium dodecyl sulfate (SDS) excipients in the shell existed in the amorphous state. This is believed to be a result of second-order interactions between the components; these could be observed by Fourier transform infrared spectroscopy. *In vitro* dissolution and permeation studies showed that the microparticles rapidly released the incorporated quercetin within one minute, and had permeation rates across the sublingual mucosa around 10 times faster than raw quercetin.

## Introduction

Nanomedicine aims to develop structures with dimensions on the nanometer scale, leading to medical materials with properties significantly different from those prepared on larger scales [1]–[2]. One of the most popular micro-/nano- structures is the core-shell structure (with different exterior and interior), in which the shell can perform a number of functions. These include: protection of the core from the outside environment; aiding in maintaining the nanomaterial's compositional and structural integrity; preventing the core from aggregating or sintering into larger particles; controlling selective percolation of molecules in and out of the interior of the material; or, increasing solubility and/or biocompatibility [1], [3], [4]. It is often thought that the bottom-up approaches are more suitable than top-down approaches for the synthesis of core-shell structures [5]. However, coaxial electrohydrodynamic atomization processes (EHDA, including electrospinning, electrospray and e-jet printing [6], [7]) have successfully been used to generate core-shell nanostructures from the macro-scale template of a concentric spinneret, for instance in the preparation of core-sheath nanofibers by electrospinning [8]-[10].

EHDA functions by dint of the fact that liquids can readily interact with electrical energy [11]. This energy is exploited to dry and solidify micro-fluid jets, causing solvent evaporation and producing micro-/nano- size products very rapidly (often on the order of 10^−2^ s). This approach has been widely used to prepare nanofibers using electrospinning [12]–[17]. Provided favorable secondary interactions exist between them, the physical state of the components in the solutions may be propagated into the solid products without phase separation. Additionally, as a result of the very fast drying process, the macroscale structure of the spinneret is reproduced in the products on the microscale. Thus, the concentric spinneret used for coaxial electrospinning results in core/shell fibers with the components in the sheath and core fluids almost entirely confined in one region of the fiber, and minimal diffusion from one to another [18]. This has made EHDA a powerful tool for developing micro-/nano- materials for biomedical applications. A notable example involves the dissolution and permeation of poorly water soluble drugs, a long-standing challenge in pharmaceutics [19]. A range of electrospun nanofibers (both monolithic and core-sheath) have been prepared to ameliorate this commonly-encountered problem, and found to exhibit excellent performance in enhancing dissolution rates [20], [21].

Over the past few years, single fluid electrospraying has been explored in the preparation of drug-loaded systems with both sustained and rapid drug release profiles [22]–[27]. Use of a coaxial electrospraying process, in which a concentric spray head can accommodate two different liquids, results in new possibilities in the materials which can be generated. Akin to coaxial electrospinning it should permit the generation of solid products from solutions which are not processable using a single fluid method, provided that processable shell fluids are used to drive the formation of a core-shell structure. Using this process, Loscertales *et al.* first reported the preparation of microparticles with a droplet encapsulated in a polymer shell in 2002 [28]. There are many reports of coaxial electrospinning being exploited to create core-sheath nanofibers in which an electrospinnable sheath fluid encapsulates a core liquid which cannot be electrospun alone [29].

Fast-dissolving drug delivery systems (FDDS) are an alternative to conventional oral solid dosage forms [30], [31]. They enhance the efficacy of active pharmaceutical ingredients by ensuring their very rapid dissolution (within one minute) in the oral cavity. This happens immediately after contact with saliva, with neither chewing nor additional water needed for administration [32], [33]. FDDS have wide ranging applications in a variety of patients such as children and the elderly, those who are bedridden, plus those suffering from vomiting, diarrhea, rapid-onset allergic attacks or central nervous system disorders. They are also useful in localised therapies (such as local anesthetics for toothaches, oral ulcers, cold sores or teething) and increase the ease of taking medicines while traveling and for patients with restricted water intake [34]. The demand for FDDS has increased rapidly in recent years. Oral FDDS have been prepared in a range of formulations, including fast-disintegrating tablets, capsules, strips, and membranes [35]–[37].

Quercetin is a plant pigment (flavonoid) found in many plants and foods. It is used for treating diabetes and conditions of the heart and blood vessels and, and has been indicated for preventing cancer, treating chronic infections of the prostate and increasing endurance and improving athletic performance [38], [39]. In Chinese traditional medicine, quercetin is often used to prevent asthma, eliminate phlegm and relieve a cough. A fast dissolving quercetin formulation with very rapid onset of action after oral administration would be highly useful to enhance patient convenience. However, quercetin has poor solubility and a high melting point, which makes the development of such systems challenging. Very few studies into improving the dissolution of quercetin using traditional pharmaceutical protocols have been reported. It is also impossible to prepare amorphous quercetin-polymer nanocomposites directly through single fluid EHDA because there is a lack of solvent systems where both i) quercetin is sufficiently soluble for formulation development and ii) EHDA processing is possible [40].

In this paper, novel core-shell microparticles permitting the rapid dissolution of quercetin were fabricated using coaxial electrospraying. Unprocessable solutions of a polymer and quercetin were encapsulated in a processable solution comprising a polymer (polyvinylpyrrolidone, PVP) and other excipients (sodium dodecyl sulfate (SDS), and sucralose). The resultant core-shell microspheres contained multiple components with each having an individual spatial deposition; the latter significantly influence the materials' functional characteristics, and are highly tunable.

## Materials and Methods

### Materials

PVP K25 (*M_w_* = 30,000) was purchased from the Sigma-Aldrich Co. Ltd. (Shanghai, China). Quercetin (purity >98%, No. MUST-12072505) was purchased from the Beijing Aoke Biological Technology Co. Ltd. (Beijing, China). Sucralose, methylene blue, and SDS were provided by the Sinopharm Chemical Reagent Co., Ltd. (Shanghai, China). N, N-dimethylacetamide (DMAc) and anhydrous ethanol were provided by the Shanghai Shiyi Chemical Reagent Co., Ltd. (Shanghai, China). All chemicals used were analytical grade. Water was double distilled just before use.

### Coaxial electrospraying

A mixed solution of 10% (w/v) PVP and quercetin in a mixed solvent of dimethylacetamide (DMAc) : ethanol (4∶6, v∶v) was used as the core fluid. The sheath liquid consisted of 10% (w/v) PVP, 0.2% (w/v) SDS and 0.3% (w/v) sucralose in a mixed solvent of water : ethanol (0.5∶9.5, v∶v).

A homemade PVC-coated concentric spinneret was used to carry out coaxial electrospraying. Two syringe pumps (KDS 100 and KDS 200, Cole-Parmer, Vernon Hills, IL, USA) were employed to drive the shell and core fluids. A high voltage supply (ZGF 60 kV/2 mA, Shanghai Sute Electrical Co., Ltd, Shanghai, China) provided an applied voltage of 20 kV (this value was selected following extensive optimization). All electrospraying processes were carried out under ambient conditions (21°C±4°C with relative humidity 57%±4%). The resultant microparticles were collected on a metal collector wrapped with aluminum foil at a fixed distance of 15 cm from the needle tip of the spinneret. Experiments were recorded using a digital video recorder (PowerShot A640, Canon, Japan) under 11× magnification. The microparticles obtained were dried for at least 24 h at 40°C under vacuum (320 Pa) in a DZF-6050 electric vacuum drying oven (Shanghai Laboratory Instrument Work Co. Ltd, Shanghai, China) to facilitate the removal of residual organic solvent and moisture. They were then stored in a desiccator before characterization was undertaken. Details of the solutions used for EHDA and the resultant processes are listed in Table 1.

**Table 1:** Parameters used for electrospraying and details of the particulate products.

No.	Process	*C_cf_* a (w/v)	*F_s_* b (mL/h)	*F_c_* c (mL/h)	*C_q_* d (%)	Diameter (μm)
M1	Single	—	1.0	—	0	1.35±0.67
M2	Coaxial	3%	1.0	0.4	7.64	1.69±1.13
M3	Coaxial	3%	1.0	0.7	10.71	1.74±1.02
M4	Coaxial	3%	1.0	1.0	—	—
M5	Coaxial	5%	1.0	0.7	16.67	1.85±0.83

a *C_cf_* represents the drug content in core fluid.

b *F_s_* represents the flow rate of sheath fluid that consists of 10% (w/v) PVP K25, 0.2% (w/v) SDS and 0.3% (w/v) sucralose in a mixed solvent system comprising water and ethanol (0.5:9.5, v:v).

c *F_c_* represents the flow rate of core fluid that consists of 10% (w/v) PVP and quercetin in a mixed solvent of DMAc and ethanol (4:6, v:v).

d *C_q_* represents the drug content in solid products, which is calculated according to the equation: *C_q_*  =  (*F*_c_×*C*_c_)/{ [*F*_s_×(10%+0.2%+0.3%)]+ [*F*_c_×(10%+*C*_c_)]}, *F*_s_, *F*_c_, and *C*_c_ represent the flow rates of the sheath and core fluids, and the drug contents in the core fluids, respectively.

### Characterization

#### Morphology

The morphologies of the core-shell microparticles were assessed using an S-4800 field emission scanning electron microscope (FESEM, Hitachi, Tokyo, Japan). Their average sizes were determined by measuring the diameters of more than 100 particles in FESEM images using the Image J software (National Institutes of Health, Bethesda, MD, USA). The topographies of the starting materials and products were observed under cross-polarized light using an XP-700 polarized optical microscope (Shanghai Changfang Optical Instrument Co. Ltd, Shanghai, China). Transmission electron microscopy (TEM) images of the samples were taken on a JEM 2100F field-emission microscope (JEOL, Tokyo, Japan). TEM samples were prepared by fixing a lacey carbon coated copper grid on the metal collector and microparticles sprayed directly onto the grid.

#### Physical status of the components

Differential scanning calorimetry (DSC) analyses were carried out using an MDSC 2910 differential scanning calorimeter (TA Instruments Co., New Castle, DE, USA). Sealed samples were heated at 10°C min^−1^ from ambient temperature (23°C) to 350°C under a nitrogen gas flow of 40 ml min^−1^. X-ray diffraction (XRD) was performed on a D/Max-BR diffractometer (RigaKu, Tokyo, Japan) over the 2*θ* range of 5–60° using Cu K*α* radiation at 40 mV and 30 mA. Attenuated total reflectance Fourier transform infrared (ATR-FTIR) spectra were recorded on a Nicolet-Nexus 670 FTIR spectrometer (Nicolet Instrument Corporation, Madison, WI, USA) over the range 500–4000 cm^−1^ and at a resolution of 2 cm^−1^. Approximately 5 mg of the materials were placed directly on the diamond window for spectra acquisition.

#### *In vitro* dissolution and permeation tests

*In vitro* dissolution studies were carried out according to the Chinese Pharmacopoeia (2010 Ed.). Dissolution studies were undertaken following Method II, a paddle method, using a RCZ-8A apparatus (Tianjin University Radio Factory, Tianjin, China). M2 (262 mg), M3 (187 mg), M5 (120 mg), or 20 mg of crude quercetin (particle size <100 μm), were placed into 600 mL phosphate buffer (PBS, pH6.8, 0.1 M) at 37±1°C and at stirred at 50 rpm under sink conditions (*C*<0.2 *C*_s_). At predetermined time points, 5.0 mL aliquots were withdrawn from the dissolution medium and replaced with fresh medium to maintain a constant volume. After filtration through a 0.22 μm membrane (Millipore, Billerica, MA, USA) and appropriate dilution, the samples were analyzed at λ_max_  = 371 nm using a UV/vis spectrophotometer (UV-2102PC, Unico Instrument Co. Ltd., Shanghai, China). The cumulative amount of quercetin released was back-calculated from the data obtained against a predetermined calibration curve. Each experiment involved 6 replicates, and the results are expressed in percentage terms as mean ± SD.

*In vitro* permeation studies were performed using a RYJ-6A Diffusion Test Apparatus (Shanghai Huanghai Drug Control Instrument Co., Ltd., Shanghai, China). The materials under study were mounted in six Keshary-Chien glass diffusion cells surrounded by a water bath system that maintained a constant temperature of 37±0.2°C. Each cell had a diffusion area of 2.60 cm^2^ and the receptor compartment had a capacity of 7.2 mL.

Porcine sublingual mucosae obtained from a local abattoir (Baoshan Jiangwan slaughterhouse, Shanghai, China) were mounted between the donor and receptor compartments of the diffusion cells with the mucosal surface uppermost. This occurred within 2 hr of slaughtering. Each donor compartment was filled with 1 ml PBS and the hydrodynamics in the receptor compartment were maintained by stirring with a Teflon coated magnetic bead at 100 rpm. The sublingual membranes were equilibrated for 30 min before permeation tests. The M2 (1.31 mg), M3 (0.93 mg), or M5 (0.60 mg) materials, or 0.1 mg of crude quercetin particles (<100 μm), were placed on the mucosal surface. Samples (1 mL) were withdrawn from the receptor compartment at timed intervals, filtered through a 0.22 μm membrane (Millipore, Billerica, MA, USA) and absorption measured at 371 nm as before. All measurements were carried out six times.

## Results and Discussion

### The PVC-coated spinneret and coaxial electrospraying

**Figure 1:**
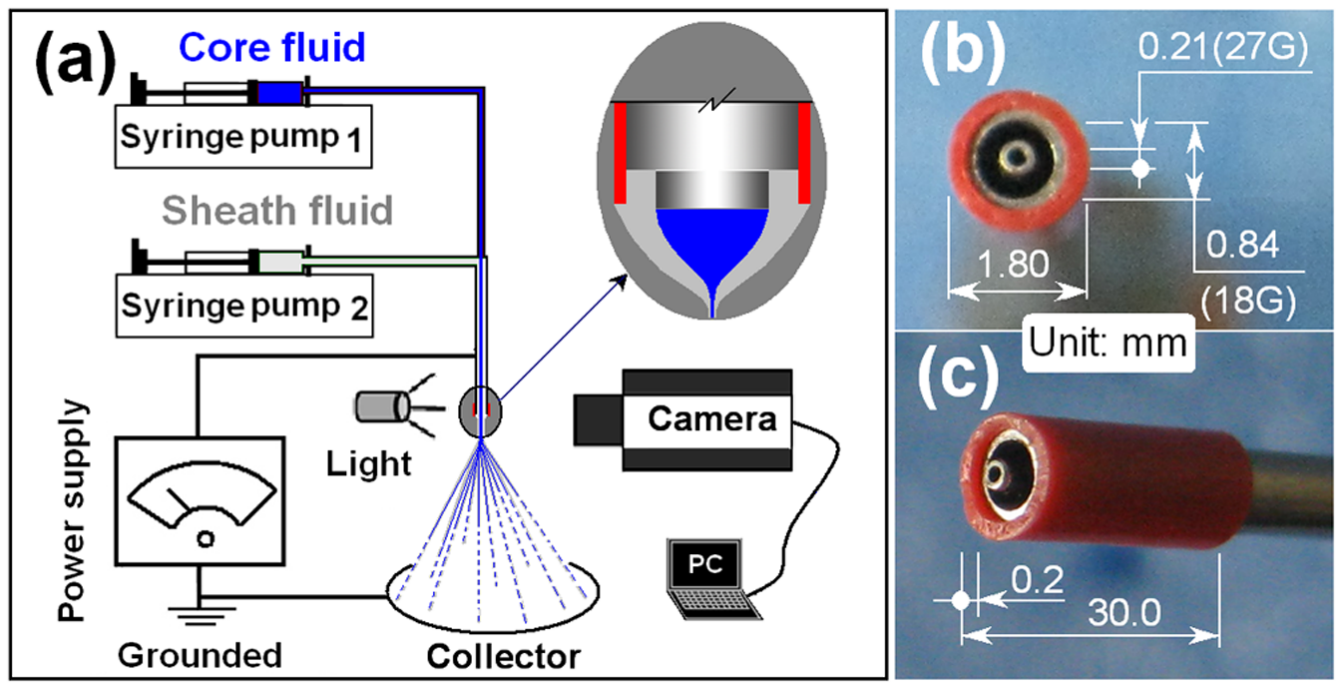
Images of the coaxial electrospraying process and the spinneret. a) a diagram of the process; b) and c) the images of the concentric PVC-coated spinneret.

Just as with electrospinning, an electrospraying system consists of four major components: one or more syringe pumps, a high-voltage power supply, a spinneret and a collector. A schematic diagram of the coaxial electrospraying process is shown in Figure 1a. The most significant modifications to the basic one-fluid process concern the spinneret, changes to which allow different procedures to be developed (e.g. coaxial electrospraying or side-by-side electrospraying for generating Janus particles [41]). Photographs of the homemade PVC-coated concentric spinneret used are included in Figure 1b and 1c. This was prepared by simply inserting a metal concentric spinneret consisting of stainless steel tubes (with inner diameters of 0.84 and 0.21 mm respectively) into a PVC tube (inner diameter 1.0 mm, length 30 mm).

**Figure 2:**
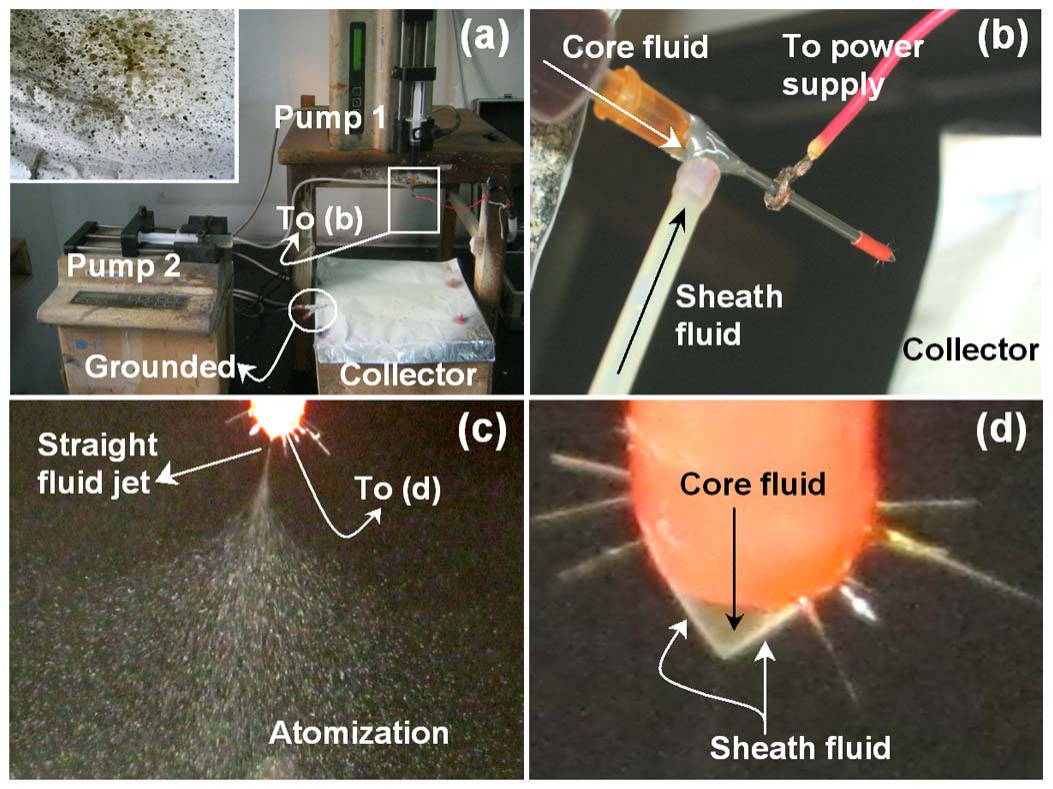
Photographs of the coaxial electrospraying processes. a) the arrangement of the apparatus used in this work (inset: a photograph of the collector when an excessive core fluid flow rate was used); b) the connection of the spinneret with the power supply; c) a typical coaxial process under an applied voltage of 20 kV, with shell and core flow rates of 1.0 and 0.7 mL h^−1^respectively; d) the compound Taylor cone.

When coaxial electrospraying was carried out, two syringe pumps were used to drive the shell and core fluids independently (Figure 2a). A copper wire was used to connect the metal part of the PVC-coated spinneret to the high voltage power supply (Figure 2b). With an applied voltage of 20 kV, and shell and core fluid flow rates of 1.0 and 0.4–0.7 mL h^−1^ respectively, a successful coaxial electrospraying process was observed (Figure 2c). A straight thinning jet is emitted from the compound Taylor cone (Figure 2d) and is followed by the explosion atomization region. Increasing the core fluid to 1.0 mL h^−1^ resulted in frequent penetration of the core solutions to the shell fluids, and destruction of the collected microparticles (M4; see inset of Figure 2a).

Quercetin not only has poor solubility in water, but also in typical organic solvents such as methanol, ethanol, chloroform and acetone. It dissolves easily in DMAc, but because of its high boiling point (166°C) PVP solutions in DMAc cannot be directly processed into solid microparticles using a single fluid electrospraying process. Thus a mixed solvent containing 40% DMAc and 60% ethanol (v/v) was selected for preparing the drug-polymer solutions. This still failed to directly generate solid products using a single fluid process, however.

Inspired by coaxial electrospinning, where an electrospinnable sheath fluid is often exploited to encapsulate an unspinnable core fluid [29], we developed here an analogous coaxial electrospraying process. In this, the shell fluid could be processed by single fluid spraying and acted as a guide surrounding the (unsprayable) core. A shell fluid consisting of 10% (w/v) PVP, 0.2% (w/v) SDS and 0.3% (w/v) sucralose in a mixed solvent of water : ethanol (0.5∶9.5, v∶v) was able to ensure a smooth coaxial electrosraying process and led to the formation of core-shell microparticles.

The PVC coating on the spinneret facilitates coaxial electrospraying in two respects: 1) being an antistatic polymer, PVC can effectively retard the loss of electrical energy from the working fluids to the environment and thus exploits the electrical field more effectively; 2) the reduced interfacial intention between the PVC and the shell working fluid (cf. between the metal spinneret and fluid) makes it easier for the compound Taylor cone to form and the straight fluid jets to be emitted. Recent reports have demonstrated similar results in electrospinning using a Teflon-coated spinneret [8] and in single fluid electrospraying using epoxy resin [42].

### Morphology

**Figure 3:**
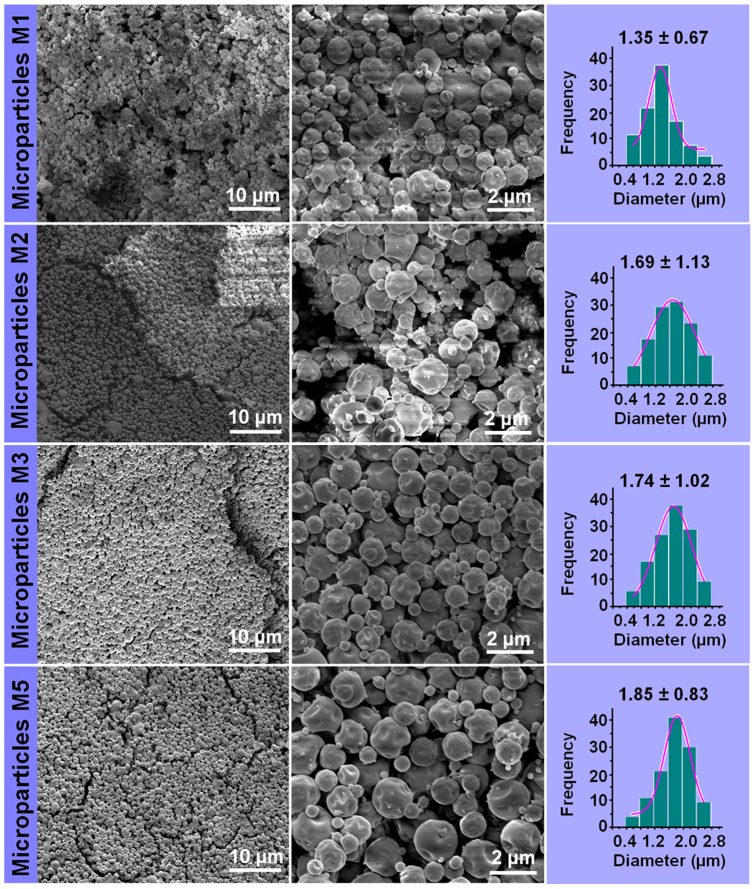
FESEM images of the microparticles and their diameter distributions.

Single fluid electrospraying of the core fluid alone did not result in solid products because of the high boiling point of DMAc, whereas spraying the shell solution alone resulted in the microparticles M1. These have an average diameter of 1.35±0.67 μm (Figure 3). With core and shell flow rates of 0.4 or 0.7 and 1.0 mL h^−1^, the core-shell microparticles M2 and M3 were generated. These respectively have average diameters of 1.69±1.13 μm and 1.74±1.02 μm (see Figure 3). This suggested that the drug content in the microparticles could be varied by increasing the flow rate of the drug-containing core fluid. However, when the core flow rate was increased to 1.0 mL h^−1^ in the preparation of M4, the core and shell fluids were found to blend, resulting in the products collected being of poor quality.

Another route to increase the amount of drug in the microparticles is to increase the drug concentration in the core fluids; M5 was thus prepared by increasing the quercetin content from 3% to 5% (w/v) and using a core flow rate of 0.7 mL h^−1^. This raised the loading from 10.71% w/w (M3) to 16.67%. The M5 particles have an average diameter of 1.85±0.83 μm (Figure 3). It is clear that an increase in core fluid flow rate from 0.4 to 0.7 mL h^−1^ and the increase of drug concentration only slightly enlarges the resultant microparticles.

**Figure 4:**
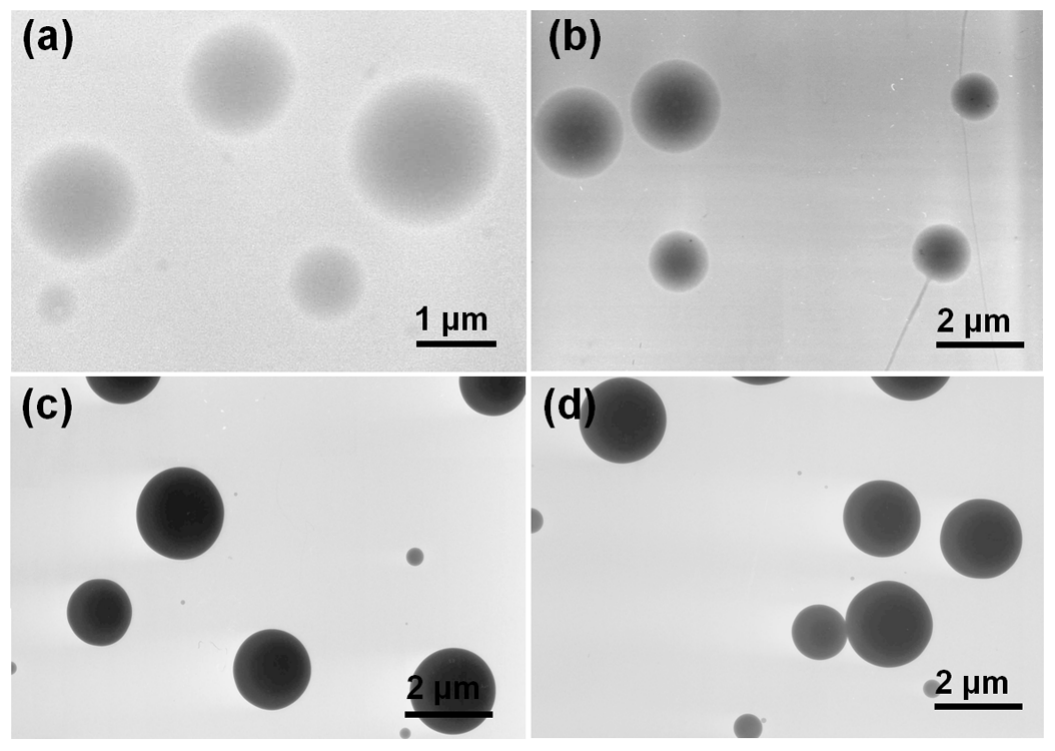
TEM images. a) M1; b) M2; c) M3; and d) M5.

Figure 4 shows TEM images of the electrosprayed products. The M1 particles (from single fluid electrospraying) are observed to be monolithic. The other three samples have clear core-shell structures, with the shells a darker gray color because of the presence of quercetin. The M2 particles have a thicker shell than the M3 and M5 materials, as expected given the reduced core fluid flow rate used to prepare the former. There is uniform gray shading in both the shell and core parts of the microparticles in all the images, indicating that quercetin is uniformly distributed in the PVP matrix in the core and that SDS and sucralose are distributed homogeneously in the shell.

### Physical form of the components

To improve the dissolution properties of poorly water soluble drug, an amorphous physical form is favored [43]. Quercetin has a very high melting point of 326°C, and thus there are few reports on the generation of amorphous forms of this drug using traditional melt and solvent evaporation methods. Similar to electrospinning [10], electrospraying is an inherently appropriate method for preparing amorphous dosage forms because of its extreme drying speed. This is able to ‘freeze’ the drug molecules randomly in the solid polymer matrix, maintaining them as a solid solution comparable to the liquid form. If there exist stabilising secondary interactions between the drug and the polymer matrix, the amorphous drug can be easily fabricated and stored for prolonged periods of time without relaxation to a crystalline form.

**Figure 5:**
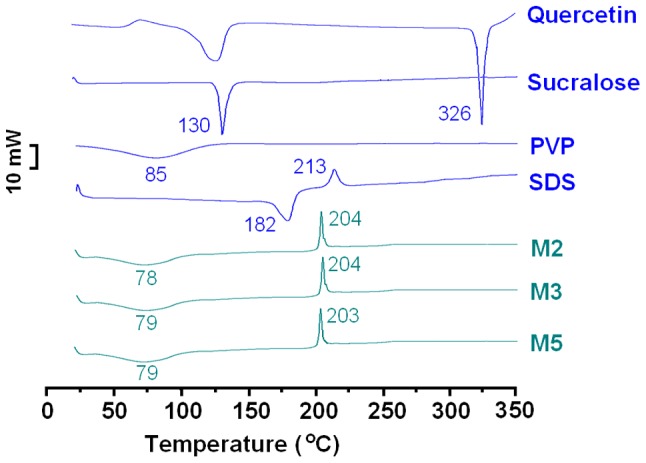
DSC thermograms of the starting materials (quercetin, sucralose, PVP and SDS) and microparticles (M2, M3 and M5).

Differential scanning calorimetry (DSC) and X-ray diffraction (XRD) experiments were undertaken to determine the physical form of the components in the microparticles. The DSC thermograms are shown in Figure 5. The DSC curves of pure quercetin and sucralose both exhibit a single endothermic response corresponding to their melting points of 130 and 326°C respectively. SDS has a melting point of 182°C followed by a decomposition temperature of 213°C. Being an amorphous polymer, PVP K25 does not show any fusion peaks or phase transitions but does exhibit a broad endotherm due to dehydration; this lies between 80–120°C with a peak at 85°C. The DSC thermograms of the three microparticle samples do not exhibit any melting peaks. They instead show broad endotherms ranging from *ca.* 60 to 100°C as a result of dehydration. The decomposition bands of SDS are narrower and sharper than that of pure SDS, and the exotherm maxima are shifted to lower temperatures. These results suggest that quercetin, SDS and sucralose are converted into the amorphous state in their respective parts of the core-shell microparticles.

**Figure 6:**
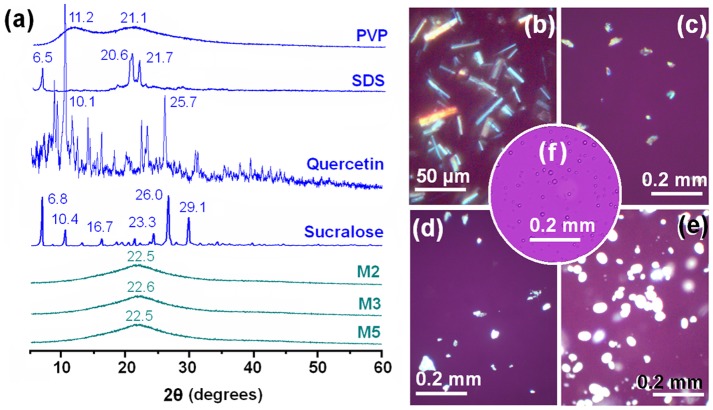
XRD patterns and cross-polarised light observations. a) XRD patterns of the starting materials and microparticles; and, microscopy images of b) quercetin; c) sucralose; d) SDS; e) PVP and f) M5 viewed under cross-polarised light.

As shown in Figure 6a, the presence of numerous distinct reflections in their XRD patterns proves that quercetin, sucralose and SDS exist as crystalline materials. This is also demonstrated by the observation of colorful images when these materials are viewed under polarized light (Figure 6b to 6d). The PVP diffraction pattern exhibits a diffuse background pattern with two diffraction halos consistent with it being an amorphous polymer. The M2, M3 and M5 particles exhibit none of the characteristic reflections of the starting materials; instead their patterns comprise diffuse haloes. In contrast to the observations of quercetin, SDS and sucralose under polarized light, the analogous images of PVP and M5 show no bright colors, suggesting both are amorphous (Figure 6e and 6f). The combined DSC, XRD and light microscopy results clearly verify that quercetin and the excipients (sucralose and SDS) exist in the amorphous form in the electrosprayed microparticles.

### Secondary-interactions between components

**Figure 7:**
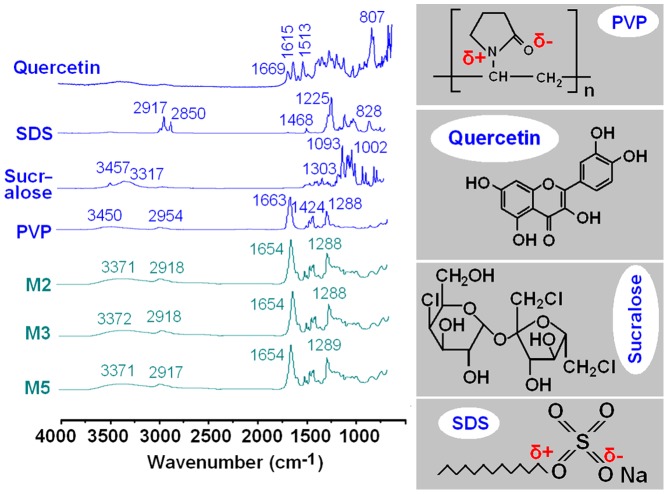
ATR-FTIR spectra of the raw materials and microparticles, and the molecular structures of PVP, quercetin, sucralose and SDS.

Compatibility among the components is essential for producing high quality and stable composite microparticles. Often second-order interactions such as hydrogen bonding, electrostatic interactions, and hydrophobic interactions can improve compatibility. The molecular structures of the four components are given in Figure 7. Quercetin and sucralose molecules possess free hydroxyl groups which could act as potential proton donors for hydrogen bonding. SDS has S = O groups and PVP has carbonyl groups, and these could act as proton receptors. Therefore it can be postulated that hydrogen bonding will occur within the core-shell microparticles.

Quercetin molecules have both –OH groups and –C = O groups, and so hydrogen bonds can form between drug molecules. This can be verified from the ATR-FTIR spectra, in which sharp peaks were visible for pure quercetin at 1669 and 1615 cm^−1^ (Figure 7), representing the stretching vibrations of –C = O groups when two molecules are bound together as dimers. However, all the peaks for quercetin were absent in the spectra of the core-shell microparticles. Only a single broad peak at 1654 cm^−1^ can be identified for M2, M3, and M5. This indicates that hydrogen bonding occurs between the PVP carbonyl group and the hydroxyl group of the quercetin molecule.

The disappearance of quercetin –C = O group bands, the shift to lower wavenumbers of peaks assigned to the C = O stretching vibrations in PVP (from 1663 cm^−1^ to 1654 cm^−1^), and the reduced intensities of some peaks in the fingerprint region of the quercetin spectrum clearly demonstrate that hydrogen bonding exists in the microparticle samples.

The SDS C–H stretching bands at 2917 and 2850 cm^−1^ and the PVP C–H stretch at 2954 cm^−1^ are not present in the microparticles' spectra. However a broader and weaker band at 2918 cm^−1^ or 2917 cm^−1^ can be seen in the spectra of M2, M3, and M5. This is indicative of hydrophobic interactions between the PVP and SDS molecules. Although electrostatic interactions cannot be observed in the ATR-FTIR spectra, they must exist between the negatively charged SDS head group and the δ+ nitrogen atom on the pyrrolidone ring of PVP [44] and also between the PVP oxygen and the electron poor C-1′ of SDS [45], as shown in Figure 7.

Based on the IR data, it is thus postulated that a combination of hydrogen bonding, hydrophobic and electrostatic forces provide a high degree of compatibility between the components that make up the core-shell microparticles. This in turn creates a homogeneous structure which will enhance the stability of the composites and ensure an even dispersion of the drug, thereby facilitating the dissolution of quercetin from the microparticles.

### *In vitro* dissolution properties

**Figure 8:**
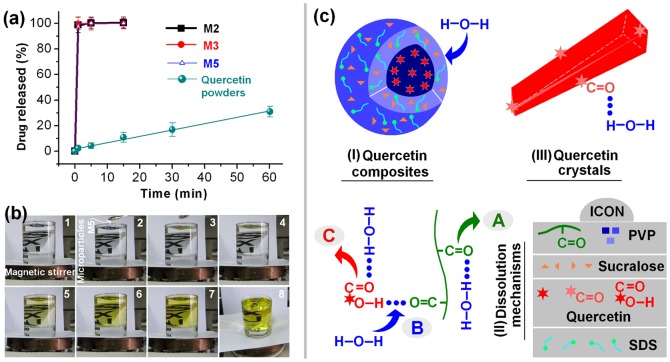
*In vitro* dissolution tests. a) *in vitro* quercetin release profiles (n = 6); b) Photographs of the dissolution of M5. The process is shown in sequence from 1 to 8, and finishes within 10s; c) a schematic depicting possible drug release mechanisms from the crystal quercetin and the microparticle composites.

Quercetin has a UV absorbance peak at λ_max_  =  371 nm; thus, the amount of quercetin released from the microparticles can be determined by UV spectroscopy using a predetermined calibration curve: *C* =  15.95*A* – 0.0017 (*R^2^* = 0.9997), where *C* is the quercetin concentration (μg mL^−1^) and *A* is the solution absorbance at 371 nm (linear range: 2 μg mL^−1^ to 20 μg mL^−1^). The measured content of quercetin in all the microparticles was equivalent to the calculated value, suggesting no drug loss during electrospraying. The *in vitro* drug release profiles of M2, M3 and M5 are provided in Figure 8a. As anticipated, all the microparticles exhibit extremely rapid release of the incorporated quercetin, freeing all the drug within one minute. The results of an experiment to observe visually the dissolution of quercetin from M5 are shown in Figure 8b. The dissolution process is shown in sequence from 1 to 8. The time taken between the addition of 50 mg of M5 to 200 mL of dissolution medium and the formation of a uniform yellow colored medium is less than 10s. In comparison, the crude quercetin particles (size ≤100 μm) dissolved only slowly, reaching 30.4% release in one hour.

A schematic depticting the proposed drug release mechanisms is given in Figure 8c. The core-shell microparticles have several key properties allowing them to improve the dissolution rates of poorly water-soluble drugs. First, the shell consists of PVP, SDS and sucralose. All have good solubility in water. In particular SDS, being a surfactant, not only facilitates the electrospraying process through reducing the surface tension of the shell fluids, but also enhances the hydrophilicity and wettability of the microparticles. This promotes their rapid disintegration (diagram (I) in Figure 8c). Second, the PVP matrix has hygroscopic and hydrophilic properties, and PVP–water interactions (“A” in Figure 8c (II)) are stronger than the polymer–drug attraction forces (“B” in Figure 8c (II)). Thus, the polymer chain can absorb solvent molecules rapidly, increasing the volume of the polymer matrix and allowing the polymer chains to loosen and uncoil. Water molecules in the dissolution medium will replace the quercetin molecules H-bonded to the –C = O groups of PVP (“B” in Figure 8c (II)), freeing the drug into solution. Quercetin molecules will also form hydrogen bonds with water (“C” in Figure 8c (II)) but this effect is very weak, as reflected by the drug's poorly water solubility.

In addition, the microparticles have small diameters and high surface to volume ratios, which means that there is a large surface area through which the drug can come into contact with the dissolution medium. Finally the drug and the matrix polymer formed amorphous composites, thereby removing the crystal lattice energy that must be overcome for dissolution of the crude quercetin powder. The latter is expected to dissolve through gradual erosion of quercetin molecules from the crystal surfaces (part (III) of Figure. 8c).

### *In vitro* permeation properties

**Figure 9:**
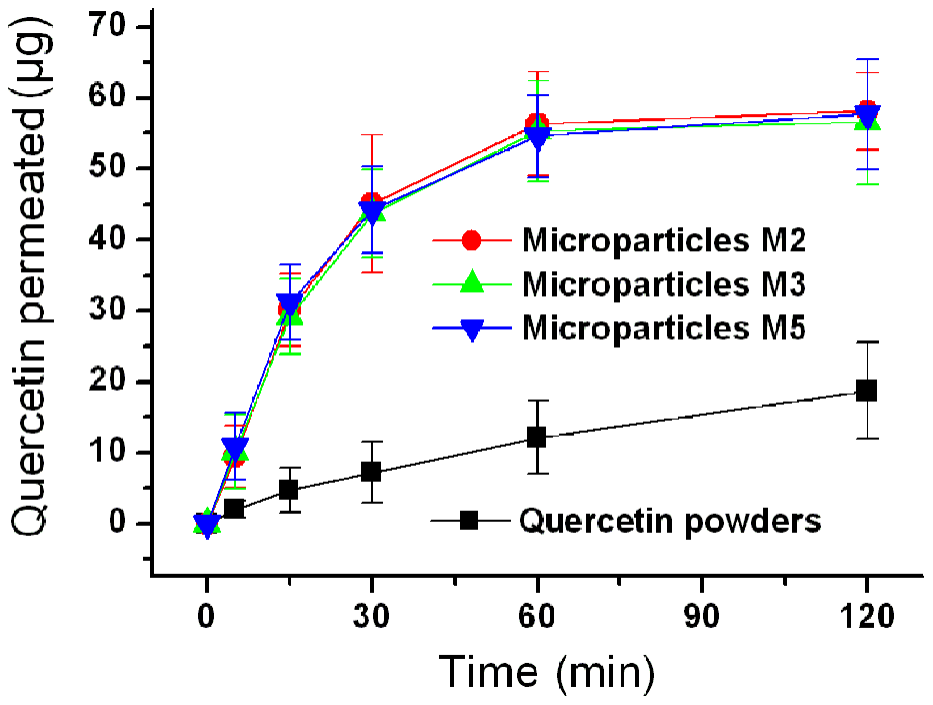
*In vitro* permeation profiles of the quercetin powder and the microparticles (n = 6).

Comparisons of the *in vitro* permeation profiles of quercetin from the microparticles and the pure powder are given in Figure 9. The cumulative permeation percentages after 30 min for M2, M3, M5 and quercetin powder were 45.1%, 43.7%, 44.2% and 7.2%, respectively. Regression values from linear release equations were estimated and found to be as follows: Q_M2_  =  1.5124t + 2.2453 (R = 0.9844, t≤30 min), Q_M3_  = 1.4515 t+2.6071 (R = 0.9847, t≤30 min), Q_M5_  = 1.4676t+3.2051 (R = 0.9764, t≤30 min) and Q_quercetin_ = 0.1501t+1.6889 (R = 0.9841, t≤120 min), where Q is the total permeation of quercetin in μg and t is the time in minutes. The particles and quercetin powder had apparent permeation rates of 0.5817, 0.5583, 0.5645 and 0.0577 μg min^−1^ cm^−2^, respectively.

The microparticles yielded a permeation rate around 10-fold faster than the quercetin powder. This is expected to be because permeability across the sublingual mucosa is a passive diffusion process, and hence faster dissolution of quercetin leads to an increased concentration gradient of drug at the mucosal surface. This facilitates rapid partitioning of drug into the sublingual mucosa and subsequent permeation. The SDS in the shell of the microparticles should also improve permeation by extracting some of the intercellular lipids which can act as rate-limiting barrier to the drug transport [46].

Although the composite microparticles M2, M3 and M5 have slightly different sizes, this difference seems to exert little influence on the *in vitro* dissolution and permeation rates of quercetin. This is probably in large part because the size difference is small, but in addition there is a homogeneous and amorphous distribution of quercetin, and the PVP which comprises the bulk of the particles is extremely hydrophilic. This results in the drug dissolving synchronously with the polymer carrier, which is a very raid process, and hence small differences in particle size do not affect dissolution or permeation markedly. By the way, sucralose is approved by FDA and is applied as a general-purpose sweetener in foods, beverages, dietary supplements and medical foods [47]. The presence of sucralose in the particle shells should thus provide an acceptable taste for patients.

## Conclusions

New fast-dissolving core-shell composite microparticles containing quercetin were successfully fabricated using coaxial electrospraying with a novel PVC-coated concentric spinneret. The product cores consisted of PVP/quercetin and the shells of PVP/SDS/sucralose. Although the quercetin-loaded solutions comprising the particle cores were unprocessable using single fluid electrospraying, the fact that the shell fluids could be processed ensured successful coaxial spraying. The drug content in the products can be tuned through the flow rate of the core solutions (within an appropriate range) or the drug concentrations therein. Particles were prepared with three different drug loadings of 7.64, 10.71, and 16.67% w/w. These had average diameters of 1.69±1.13, 1.74±1.02 and 1.85±0.83 μm respectively. SEM and TEM images clearly evidence the core/shell structure. The components in both the core and the shell exist in an amorphous physical form as a result of favorable interactions between them, as demonstrated by XRD, DSC and ATR-FTIR. The microparticles increased both the dissolution and permeation rates of quercetin. Such materials thus have the potential to enhance the dissolution and permeation performance of poorly water-soluble drugs, and to be further developed as novel oral or sublingual drug delivery systems.
